# Cross-π-conjugated enediyne with multitopic metal binding sites†

**DOI:** 10.1039/d0ra06320g

**Published:** 2020-10-20

**Authors:** Djawed Nauroozi, Benjamin Wurster, Rüdiger Faust

**Affiliations:** Institute for Chemistry, CINSaT – Centre for Interdisciplinary Nanostructure Science and Technology, University of Kassel Heinrich-Plett-Str. 40 34132 Kassel Germany r.faust@uni-kassel.de; Institute for Inorganic Chemistry I, Ulm University Albert-Einstein-Allee 11 89081 Ulm Germany djawed.nauroozi@uni-ulm.de

## Abstract

The synthesis of an enediyne molecule functionlized with different metal coordination sites in a cross-π-conjugated fashion is reported. Using Pd-mediated cross-coupling reactions, 2,2′-bipyridine units were attached at the periphery of diazafluorenemethylidene to obtain a multitopic ligand. UV-vis spectrosopic investigations along with electrochemical analyses reveal electronic communication along the conjugated path reflected in red-shifted absorption spectra and shifts of reduction potentials. The properties of the ligand could be manipulated by coordinating [Ru(bpy)_2_]^2+^ fragments at all three coordination spheres of the molecule while the different complexing imine moieties serve as possible coordination sites for various metal centres.

## Abstract 2

Synthesis and structural characterization of a geminal enediyne molecule with three imine metal binding sites based on diazafluorenemethylidene in a cross-π-conjugated fashion is reported.

## Graphical abstract

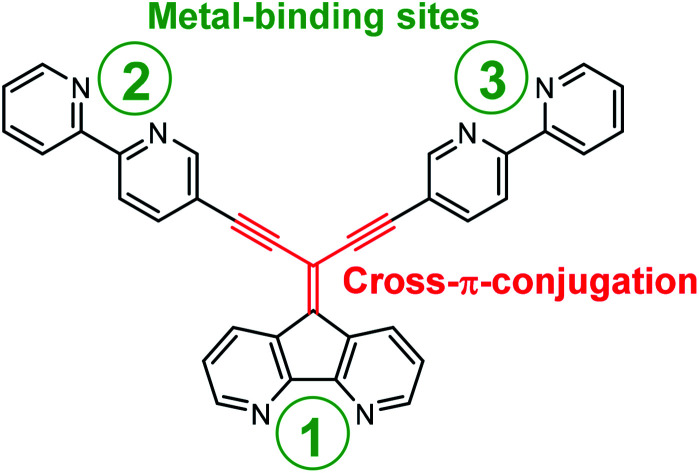

## Introduction

Well-defined molecular architectures comprising several metal binding sites are of continuing interest as they can be used for solar cell applications,^1–4^ photocatalytic solar fuel production,^5–7^ molecular electronics^8^ and biomedical applications such as photodynamic therapy.^8,9^ In this context, 2,2′-bipyridine and the corresponding Ru(ii) complexes have been widely used in various π-as well as non-conjugated moieties^10^ while the rich coordination chemistry serves as a prerequisite for 2D and 3D coordination complexes of manifold geometries.^11–14^

The design of the ligand in supramolecular architectures usually dictates the supramolecular arrangement^15–17^ as well as the electrochemical and photophysical properties such as the directionality of the electron and energy transfer, excited state lifetimes and interaction modes of the complexes with other interconnecting molecules.^18–20^

As the majority of literature-known systems provide metal bindings sites embedded in a linear π-conjugated fashion,^21–24^ we proposed an alternative design, namely the cross-conjugated approach with an enediyne moiety attached to a diazafluorene building block. The principal design of our approach based on a dibromoolefin building block^25^ allows for simple functionalization at the ene moiety as demonstrated recently with various protecting groups,^26^ bulky end-caps^26^ or redox-active fragments.^27^ The effective electronic communication observed in related architectures led us to design a molecular ligand that can serve as starting building block for large supramolecular ensembles.

**Fig. 1:**
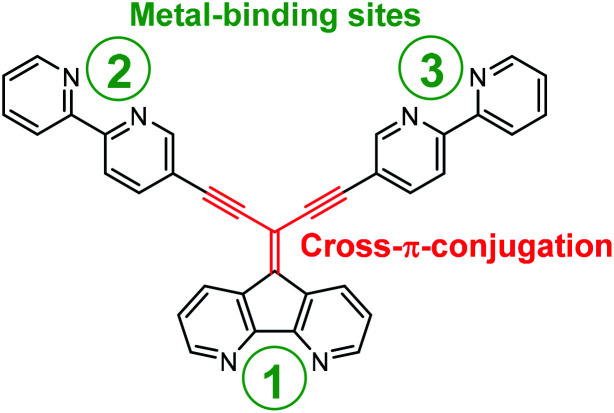
Illustration of a cross-π-conjugated ligand based on diazafluorene core, decorated with 2,2′-bipyridine complexing units at the periphery.

In this report we use the diazafluorene core to access a cross-π-conjugated ligand with multitopic metal binding sites of different nature realized by introduction of 2,2′-bipyridines into the periphery of the diazafluorene unit (Fig. 1).

## Results and discussion

### Syntheses

**Scheme 1:**
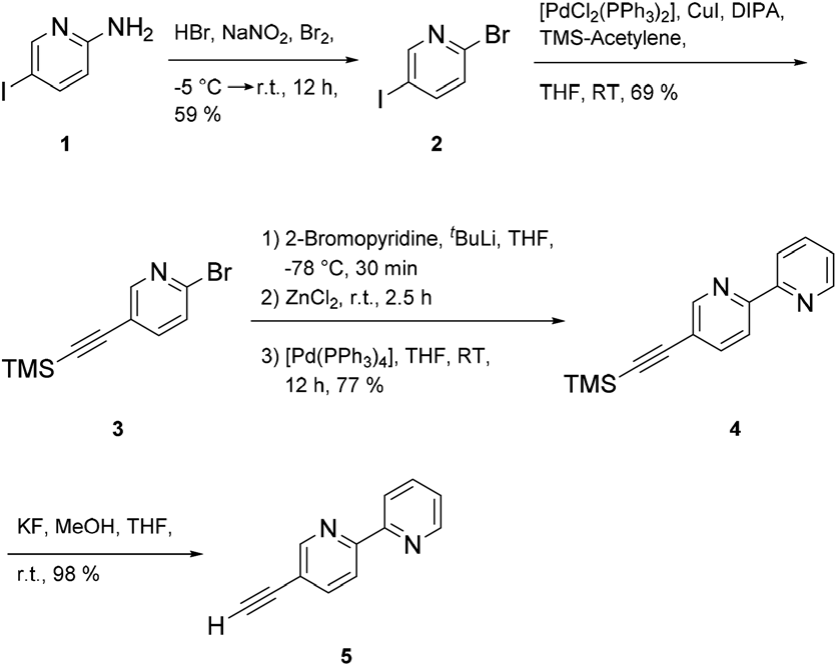
Synthetic sequence of 5-(ethynyl)-2,2′-bipyridine 5.

Synthesis of the multitopic N-heterocyclic ligand required access to 5-ethynyl-2,2′-bipyridine 5 (ref. 28) as a prerequisite for additional metal binding site. Starting with 5-amino-2-iodopyridine 1 the amine group was converted to the bromo functionalized species *via* a Sandmeyer reaction. Chemoselective coupling of 2 (ref. 29) with trimethylsilyl acetylene (TMS-acetylene) gave 3, which in turn could be used in a Negishi reaction to deliver 4 (ref. 28) in good yields (77%). Protodesilylation of 4 provided 5-(ethynyl)-2,2′-bipyridine 5 in almost quantitative yields using potassium fluoride as a fluoride source in protic THF (Scheme 1).

**Scheme 2:**
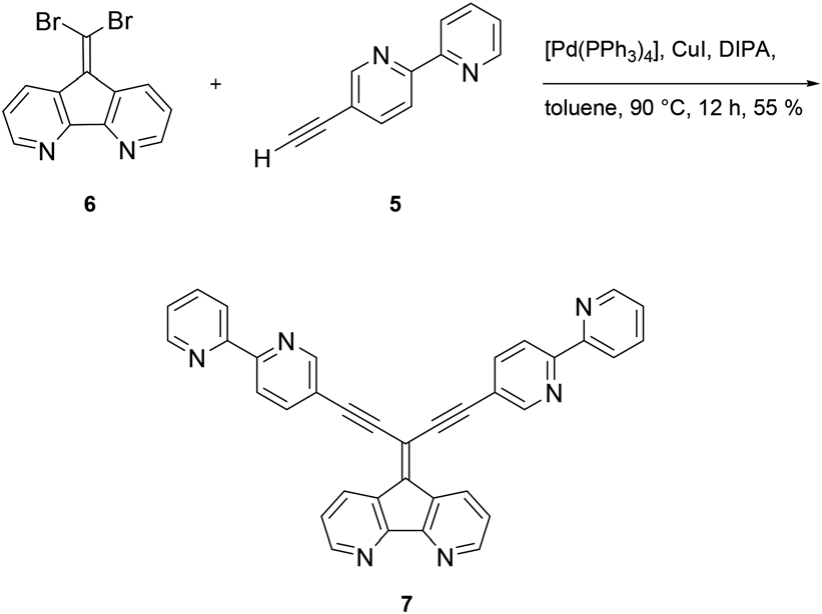
Synthesis of ligand 7*via* Sonogashira–Hagihara coupling reaction.

Formation of ligand 7 was accomplished under Sonogashira coupling conditions between ethynylbipyridine 5 and dibromoolefin 6,^25^ which was developed recently in our labs (Scheme 2). Purification of 7 proved to be tedious due to strong interaction of 7 with the stationary phases when using chromatography methods. Hence, purification of the compound was accompanied with loss of the product. Other methods such as precipitation, crystallization or sublimation in order to purify the compound all were not successful. The low solubility of 7 in most organic solvents posed a challenge regarding spectroscopic characterization of the compound. While NMR measurements in separate deuterated organic solvents all failed, only a mixture of chloroform/methanol (1 : 2/v/v) gave satisfactory ^1^H and ^13^C NMR spectra (see ESI Fig. S1 and S2†). However, along with spectroscopic characterization, the constitution of 7 was confirmed by elemental analysis as well as by high resolution mass spectrometry (see ESI Fig. S3 and S4†).

**Scheme 3:**
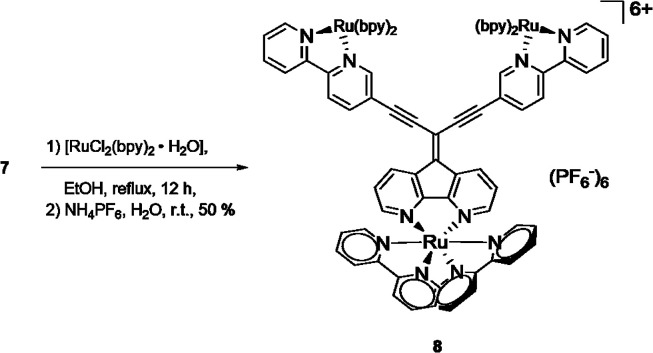
Synthesis of triply complexed ligand 7.

The potential of metal complexation of 7 at all three coordination sites was approached by refluxing a mixture of [Ru(bpy)_2_Cl_2_] and 7 in ethanol (Scheme 3). After a reaction time of 12 h, the cooled mixture was treated with an aqueous solution of NH_4_PF_6_ and complex 8 could be obtained after filtration as a red precipitate with the hexafluorophosphate counter ion. Similar to the corresponding ligand 7, the solubility of 8 in usual organic solvents was poor. However, ^1^H NMR analysis of the complex in deuterated MeOH revealed expected set of signals for the complex, although broad signals suggest a mixture of isomers (ESI, Fig. S2†). Meaningful ^13^C NMR data of the complex could not be obtained despite extended recording times. However, elemental analysis and high resolution mass spectrometry (ESI, Fig. S5–S7†) confirmed the constitution of the triply Ru-complexed compound.

### Photophysical properties

**Fig. 2:**
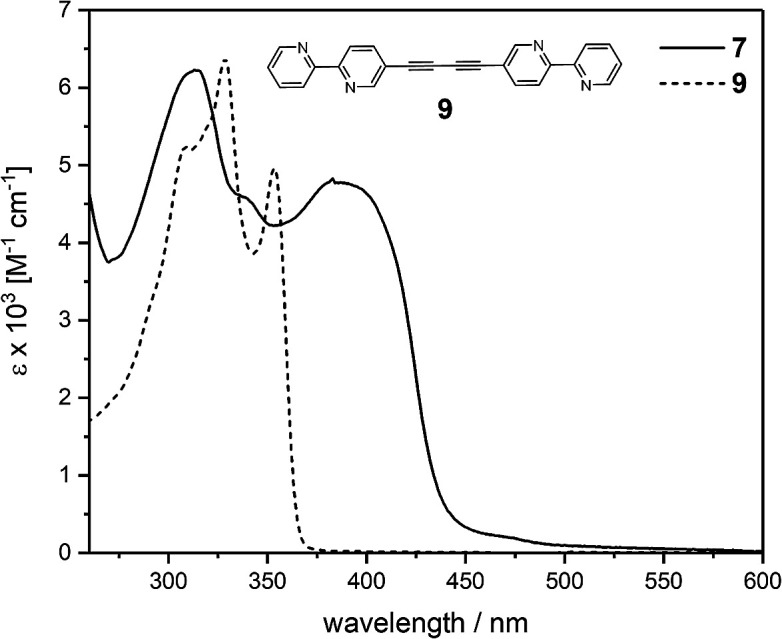
UV-vis absorption spectra of ligand 7 and the linear conjugated counterpart 9 in CH_2_Cl_2_ at r.t.

**Fig. 3:**
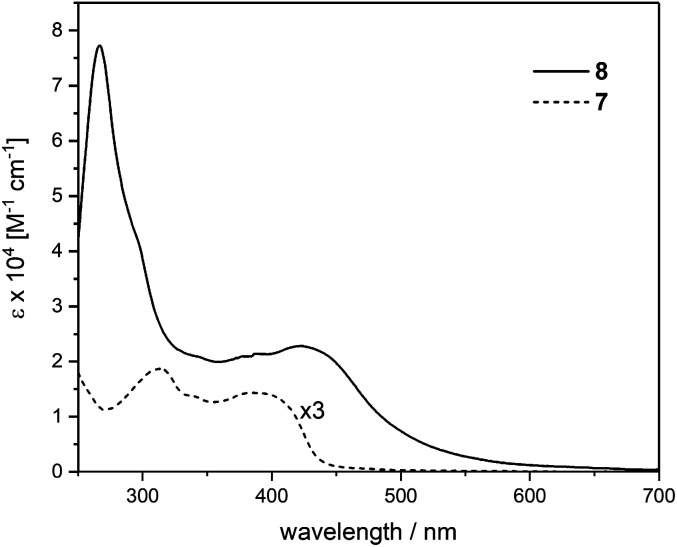
UV-vis absorption spectra of ligand 7 (magnified) and the corresponding Ru complex 8, measured in CH_2_Cl_2_ at r.t.

The photophysical properties of ligand 7 and the corresponding complex 8 were investigated in dichloromethane solutions of each compound (Fig. 2 and 3). The pertinent data are collected in Table 1. The absorption spectrum of 7 ranges between 265 and *ca.* 500 nm with absorption maxima at 314 and 388 nm. The shortest wavelength absorption maximum presumably arises from ethynylbipyridine centered transitions, as suggested by comparison of the spectral profile of this fragment (see ESI Fig. S8†). Introduction of the aryl-substituted alkyne termini towards assembling cross-π-conjugated ligand systems and, hence, an increase of the conjugation path, are reflected in a red-shift of the longest wavelength absorption maximum at 388 nm. Interestingly, π-electron delocalization in this particular cross-conjugated system seems to be even more efficient when compared with the absorption features of the parent linear π-conjugated bis(ethynylbipyridine) 9 (see inset Fig. 2). Here, the absorption ranges up to only *ca.* 370 nm while the absorption of 7 exceeds 450 nm. Certainly, the bipyridine units arrange in a tilted fashion explaining the smaller absorption range for molecule 9.

**Table 1:** UV-vis data of compounds 5, 7, 8, 9 and [Ru(bpy)_2_Cl_2_], measured in CH_2_Cl_2_ solutions at r.t.

Compound	*λ* [nm]	*ε* × 10^3^ [M^−1^ cm^−1^]
5	300, 311(sh)	5, 4
7	313, 339(sh), 388	6, 5, 5
8	266, 295, 424	78, 43, 23
9	308, 329, 353	5, 6, 5
[Ru(bpy)_2_Cl_2_]	276, 366, 561	82, 17, 17

The UV-vis spectrum of the trinuclear complex 8 covers a range up to 650 nm and features higher intensities compared to the corresponding ligand 7 as seen in Fig. 3 with magnified spectrum of 7. The spectrum of 8 shows a pronounced ligand centered (LC) transition at 266 nm with a shoulder at 295 nm. A broad absorption peaking at 424 nm points toward a featureless metal-to-ligand charge-transfer (MLCT) as was observed in similar cross-conjugated systems reported recently.^27^ These findings suggest strong interaction between the cross-conjugated ligand system with the corresponding metal centers reflected in the extended absorption range.

### Electrochemical investigations

**Fig. 4:**
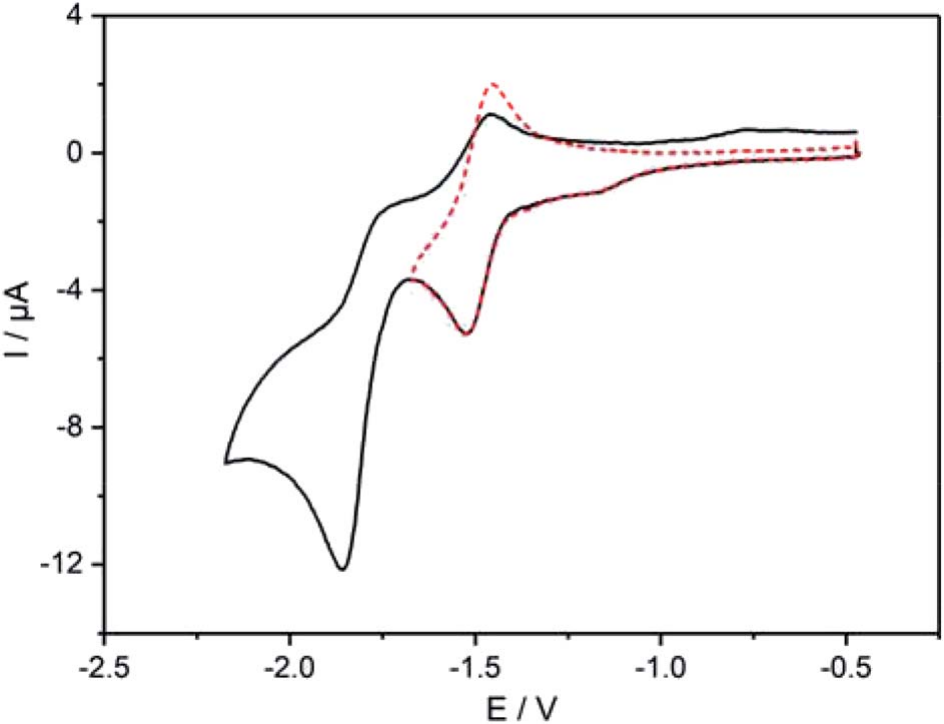
Cyclic voltammogram of 7, performed in CH_2_Cl_2_ solution at room temperature containing 0.1 M ^*n*^Bu_4_NPF_6_ with Ag/AgCl as reference electrode, Pt wire as counter electrode and Pt disk as working electrode; scan rate: 150 mV s^−1^; all data referenced against external Fc^+^/Fc.

**Fig. 5:**
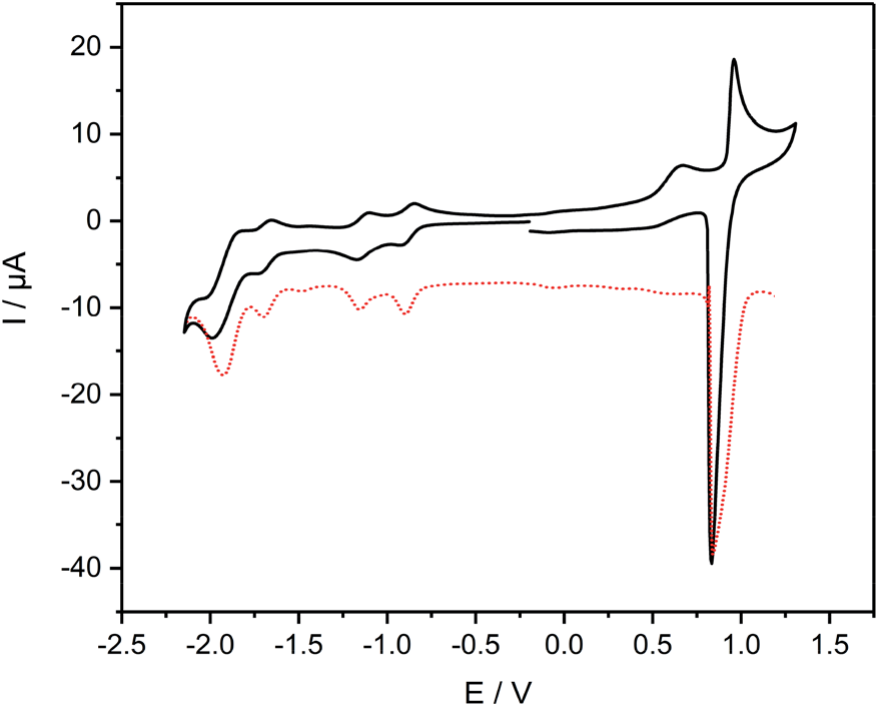
Cyclic and differential pulse voltammogram of 8, performed in CH_2_Cl_2_ solution at room temperature containing 0.1 M ^*n*^Bu_4_NPF_6_ with Ag/AgCl as reference electrode, Pt wire as counter electrode and glassy carbon as working electrode; scan rate: 100 (CV)/50 (DPV) mV s^−1^; all data referenced against external Fc^+^/Fc.

The electrochemical features of 7 and 8 were investigated by cyclic voltammetry and the complex additionally by differential pulse voltammetry in dichloromethane solutions of the compounds containing ^*n*^Bu_4_NPF_6_ as electrolyte salt (Fig. 4 and 5). The data are referenced against Fc^+^/Fc and summarized in Table 2. The cyclic voltammogram of 7 (Fig. 4) shows two reduction waves at −1.49 V and −1.81 V, respectively. The first wave is attributed to the reduction of the diazafluorene unit, which is in line with our previous findings with similar compounds.^27^ This first redox event is reversible (Fig. 4 dotted line) unless measuring beyond −1.80 V, which leads to the loss of reversibility of this event. A second quasi-reversible redox process at −1.81 V can be assigned to the reduction of bipyridine units within the molecule. The higher current intensities for this event suggest a simultaneous reduction of both bipyridine parts, which implies an intermittent electronic communication between both bipyridine units. Remarkably, the reduction potential of the bipyridines in ligand 7 are shifted cathodically by almost 300 mV compared to the reduction potential of ethynylbipyridine 5 alone (see ESI Fig. S9†). This is due to the diazafluorene unit being reduced first and consequently affecting the reducibility of the bipyridines. The observed behavior again underlines the electronic communication along the cross-π-conjugated path.

**Table 2:** Cyclic voltammetry data of 5, 7 and 8^a^

Compound	*E*_1/2red_ [V]	*E*_1/2ox_ [V]	Δ*E* [mV]
5	−1.53		
7	−1.49/−1.81		70/108
8	−0.88/−1.14/−1.68/−1.91	0.96	68/62/61/150/127

a For conditions, see Fig. 4 and 5.

The cyclic voltammogram of complex 8 (Fig. 5) reveals an oxidation event at 0.90 V that is attributed to the oxidation of the Ru^II/III^ centers. The reverse scan of this event shows an intense spike for current, which points towards adsorption processes at the electrode. The high current is observed in the differential pulse voltammogram as well (Fig. 5). Noteworthy is an irreversible wave peaking at 0.66 V that can only be observed in cyclic voltammetry measurements. Reduction of 8 reveals two consecutive reversible waves at −0.88 V and −1.14 V, respectively, that can be assigned to the reduction of both diazafluorene and ethynylbipyridine units. Remarkable is the anodic shift of more than 600 mV for both events compared to the reduction potential of the ligand 7, which underlines the electron-withdrawing nature of the Ru centers promoting the reduction of the respective ligand units. Another reversible reduction wave at −1.68 V followed by a quasireversible event at −1.91 V can be attributed to the reduction of bipyridines while the latter points towards simultaneous reduction of several bipyridine units considering the higher current detection.

The photophysical and electrochemical properties of the synthesized ligand and the corresponding Ru complex support previous findings in similar cross-π-conjugated systems. As such, ligand 7 is of possible use for the design of catalytic ensembles as it provides different binding moieties for individual metal centers, while offering electronic communication between the different units.

## Conclusions

In summary, a cross-π-conjugated ligand bearing multitopic metal binding sites is synthesized by using Pd-mediated cross-coupling reactions. UV-vis spectroscopic analyses of the ligand revealed strong interaction between the different parts of the molecule *via* the cross-conjugated system. The structure of the ligand provided access to synthesis of trinuclear complexes, which was demonstrated with coordinating [Ru(bpy)_2_]^2+^ fragments at all coordination sites. Here, the dominant interaction between the metal centres and the diazafluorene unit was reflected in the extended absorption range. The electrochemical results of both ligand and the complex suggest that the first reduction event based on the diazafluorene moiety influences the reduction ability of the complex, as a cathodic shift is observed for ethynylbipyridine parts in the ligand compared to the ethynylbipyridine molecule alone. Comparison of reduction potentials of ligand 7 and the corresponding complex 8 underlines further the effective communication along the conjugated path as an anodic shift of more than 600 mV is observed for the reduction of the diazafluorene and the ethynylbipyridine units. The different nature of metal binding sites within the molecule allows for selective coordination of metal centres, a topic of continuing interest in different fields such as photo-driven catalytic reactions.

In total, the persistent electronic communication in presented ligand design alongside the flexible synthetic modification of peripheral substituents allow for manipulating the properties of such ligand systems. In particular, the different nature of imine binding sites provided in ligand 7 paves the way to chemoselective control of coordination chemistry for various applications.

## Conflicts of interest

There are no conflicts to declare.

## Supplementary Material

RA-010-D0RA06320G-s001
