# Supplementary material for: Cross-π-conjugated enediyne with multitopic metal binding sites
Source: RSC Adv. 2020 Oct 20;10(63):38612–6. doi: 10.1039/d0ra06320g (PMC9057285; doi:10.1039/d0ra06320g)
Supplement: RA-010-D0RA06320G-s001 [file RA-010-D0RA06320G-s001.pdf]

Electronic Supplementary Material (ESI) for RSC Advances.
This journal is © The Royal Society of Chemistry 2020

Supporting Information

Cross-π-Conjugated Enediyne with Multitopic Metal Binding Sites

Djawed Nauroozi,*[a,b] Benjamin Wurster,[a] and Rüdiger Faust *[a]

[a] Institute for Chemistry and CINSaT – Centre for Interdisciplinary Nanostructure Science and Technology, University of Kassel, Heinrich-Plett-Str. 40, 34132 Kassel, Germany.

[b] Ulm University – Institute for Inorganic Chemistry I, Albert-Einstein-Allee 11, 89081 Ulm, Germany.

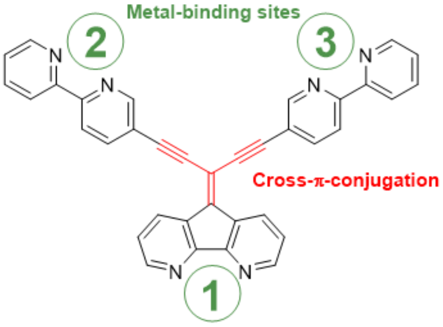

Content

I.	Synthetic procedures	page 2
II.	^{1}H and ^{13}C NMR spectra	page 4
III.	Mass spectrometry data	page 6
IV.	UV-vis of (5-ethynyl)-2,2'-bipyridine **5**	page 10
V.	Cyclic voltammetry of (5-ethynyl)-2,2'-bipyridine **5**	page 10
VI.	Differential pulse voltammogram of **8**	page 11
VII.	References	page 11

Synthetic procedures

2-Bromo-5-iodopyridine, 2-bromo-5-(trimethylsilylethynyl)pyridine, 5-(trimethylsilylethynyl)-2,2‘-bipyridine and 5-ethynyl-2,2’-bipyridine were prepared according to literature and the characterization matched with the data given therein.

*2-Bromo-5-iodopyridine (**2**)*[1]

δ = 8.57 (d, *J* = 1.92 Hz, 1H), 7.81 (dd, *J* = 8.27 Hz, 2.27 Hz, 1H), 7.27 (d, *J* = 8.40 Hz,1H)
δ = 156.15, 146.55, 141.42, 129.94, 91.68;
APCI [M+H]+: 284 m/z

*2-Bromo-5-(trimethylsilylethynyl)pyridine (**3**)*[2]

δ = 8.43 (s, 1H.), 8.59-8.54 (d, *J* =7.93 Hz, 1H), 7.45-7.40 (d, *J* =8.02 Hz, 1H), 0.26 (s, 9H);
δ = 153.15, 141.59, 141.28, 127.87, 120.01, 100.44, 100.31, 0.08;
APCI [M+H]+: 256 m/z

*5-(Trimethylsilylethynyl)-2,2‘-bipyridine (**4**)*[3]

δ = 8.72 (s, 1H), 8.65-8.68 (d, *J* = 4.3 Hz, 1H), 8.42-8.34 (m, 2H), 7.88-7.84 (dd, J = 8.3 Hz, 1.5 Hz, 1H), 7.84-7.77 (m, 1H), 7.33-7.27 (m, 1H),0.27 (s, 9H);
δ = 152.14, 149.24, 139.94, 137.20, 124.08, 121.59, 120.35, 101.93, 99.30, 0.03;
APCI [M+H]+: 253 m/z

*5-Ethynyl-2,2’-bipyridine (**5**)*[3]

δ = 8.77 (s, 1H), 8.71-8.86 (d, *J* = 4.2 Hz, 1H), 8.45-8.41 (d, 2H, *J* = 8.0 Hz), 7.93-7.89 (dd, *J* = 8.2 Hz, 1.3 Hz, 2H), 7.88-7.83 (t, *J* = 7.4 Hz, 1H), 7.37-7.32 (m, 1H); 3.30 (s, 1H)
δ = 155.50, 155.40, 152.30, 149.40, 140.10, 137.10, 124.20, 121.50, 120.40, 119.20, 81.5, 80.80;
APCI [M+H]+: 181 m/z

*9H-Bis(5-ethinyl-2,2’-bipyridin)methylen-4,5-diazafluoren (**7**)*

100 mg (300 µmol) dibromoolefin **6** and 21 mg (18 µmol) [$Pd(PPh_3)_4$] were suspended in 30 ml toluene and 8 ml di-*iso*-propylamine. 107 mg (5990 µmol) ethynylbipyridine **5** together with 6 mg (30 µmol) CuI were added to the mixture und the reaction heated up to 90 °C over night. After cooling to room temperature the mixture was hydrolysed with demineralized water and extracted with chloroform (3 x 15 ml). The combined organic phases were washed with sat. aqueous NH_4Cl solution (3 x 15 ml) and sat. NaCl solution (1 x 20 ml). The organic phase was dried over $MgSO_4$, filtered and the solvent removed under reduced pressure. The product was obtained after silica column chromatography (silica, $CHCl_3$/MeOH 20:1) as a yellow solid in 55 % yield.

δ = 8.86 (dd, *J* = 2.10 Hz, 0.81 Hz, 2 H), 8.85 (dd, *J* = 6.58 Hz, 1.39 Hz, 2 H), 8.61 (dd, *J* = 4.88 Hz, 0.52 Hz, 2 H), 8.58 (dd, *J* = 3.53 Hz, 1.40 Hz, 2 H), 8.37-8.32 (m, 4 H), 7.88 (ddd, *J* = 13.78 Hz, 6.10 Hz, 1.76 Hz, 2H), 7.41-7.34 (m, 4 H);
δ = 156.60, 155.21, 155.14, 151.62, 150.49, 148.65, 139.96, 138.21, 132.50, 132.01, 124.74, 123.75, 122.10, 120.92, 119.12, 104.41, 96.33, 91.13;
ν = 3072, 3027, 2920, 2080, 1645, 1631, 763.
HRMS / ESI (pos): Calculated [$C_{36}H_{21}N_6$] = 537.1822, experimental: 537.1834
Elemental analysis: Calculated: **C** – 80.58, **H** – 3.76, **N** – 15.66;
Experimental: **C** – 76.70, **H** – 4.02, **N** – 15.54;

*[((bpy)$_2$Ru(II))$_3$[9H-Bis(5-ethinyl-2,2'-bipyridin)methylen-4,5-diazafluoren]-hexakis(hexafluorphosphat)] (**8**)*

30 mg (56 µmol) of **7** were dissolved in 10 ml EtOH and 5 ml CH_2Cl_2. To this solution 81 mg (170 µmol) [$RuCl_2(bpy)_2$] were added and the mixture heated under reflux for 12 h. The obtained red suspension was cooled to room temperature and an aqueous solution of NH_4PF_6 was added until no precipitation was observed. The mixture was kept at -18 °C over night, the precipitation was then filtered und washed with cold water, EtOH and Et_2O. The product was obtained as a red solid in 50 % yield.

δ = 8.80-8.60 (m, 18 H), 8.35-8.25 (m, 2 H), 8.20-8.05 (m, 16 H), 8.00-7.90 (m, 8 H), 7.90-7.80 (m, 8 H), 7.78- 7.70 (m, 2H), 7.60-7.45 (m, 14 H);
ν = 3052, 3017, 2715, 1993, 1585, 1481, 796.
HRMS / MALDI (pos): Matrix (DCTB) Calculated [$C_{96}H_{70}F_{30}N_{18}OP_5Ru_3$] = 2521.1319, Experimental: 2521.1249.
Elemental analysis: Calculated: **C** – 43.57, **H** – 2.59, **N** – 9.53;
Experimental: **C** – 43.68, **H** – 2.59, **N** – 9.20;

^{1}H and ^{13}C NMR spectra

Ligand **7** ($CDCl_3$/$MeOD_3$ 1:2)

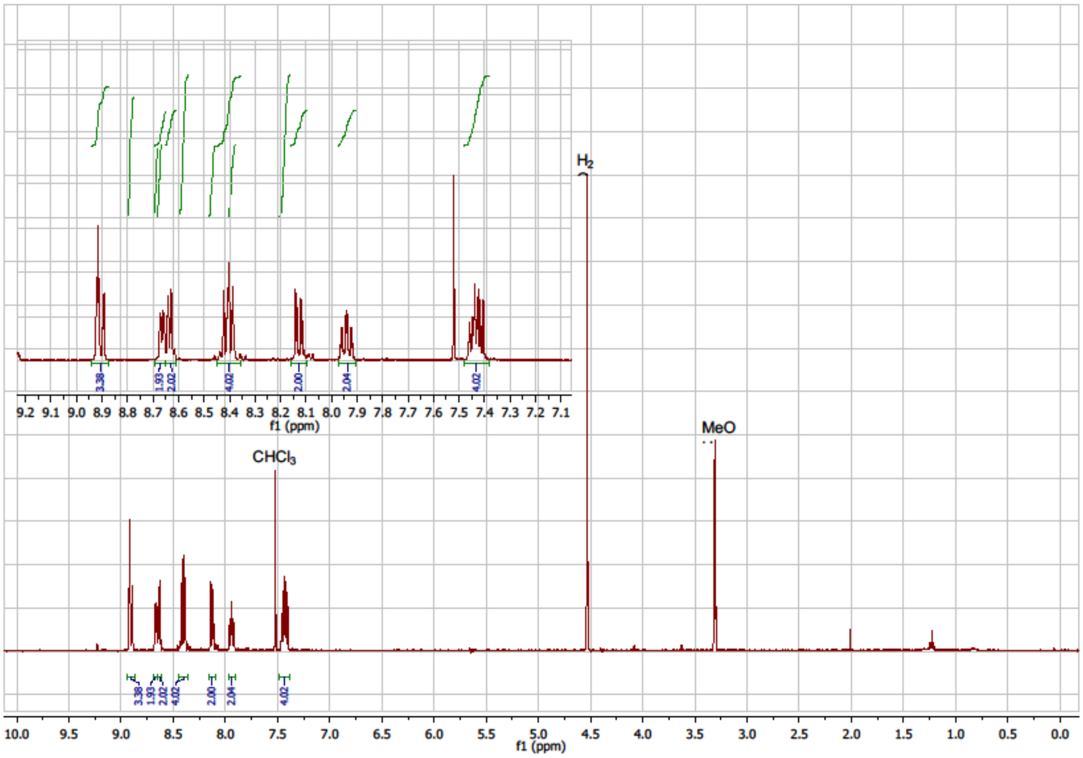

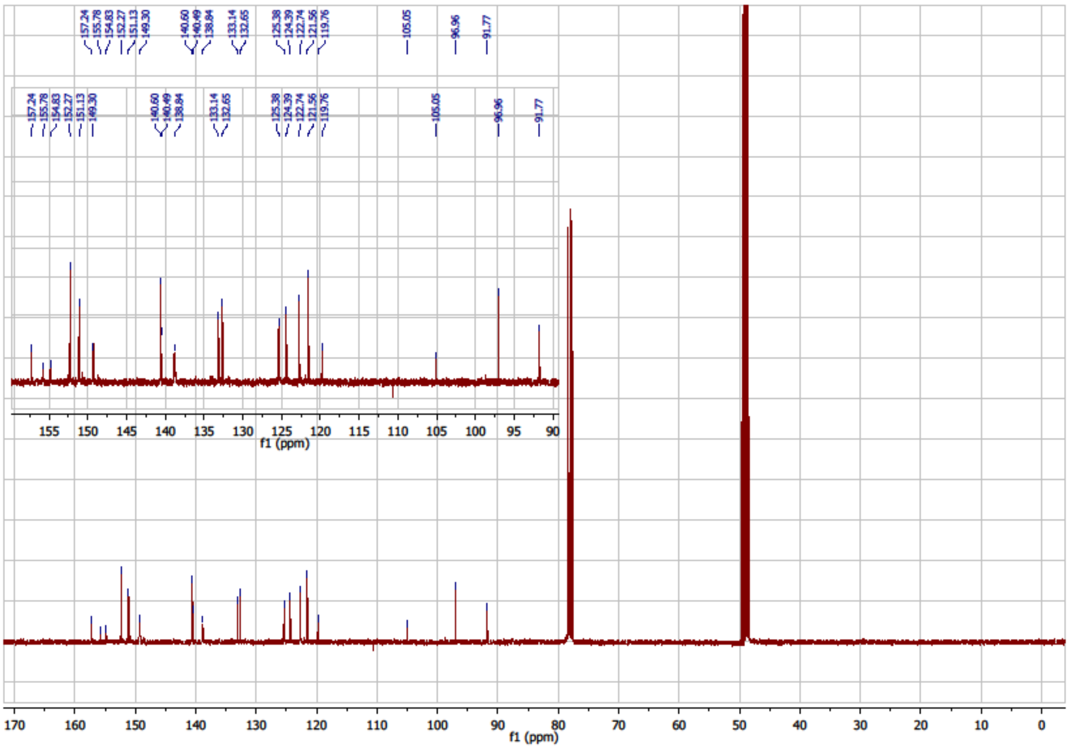

Figure S1: ^{1}H and ^{13} NMR of **7**.

^{1}H NMR of complex **8** ($MeOD_3$)

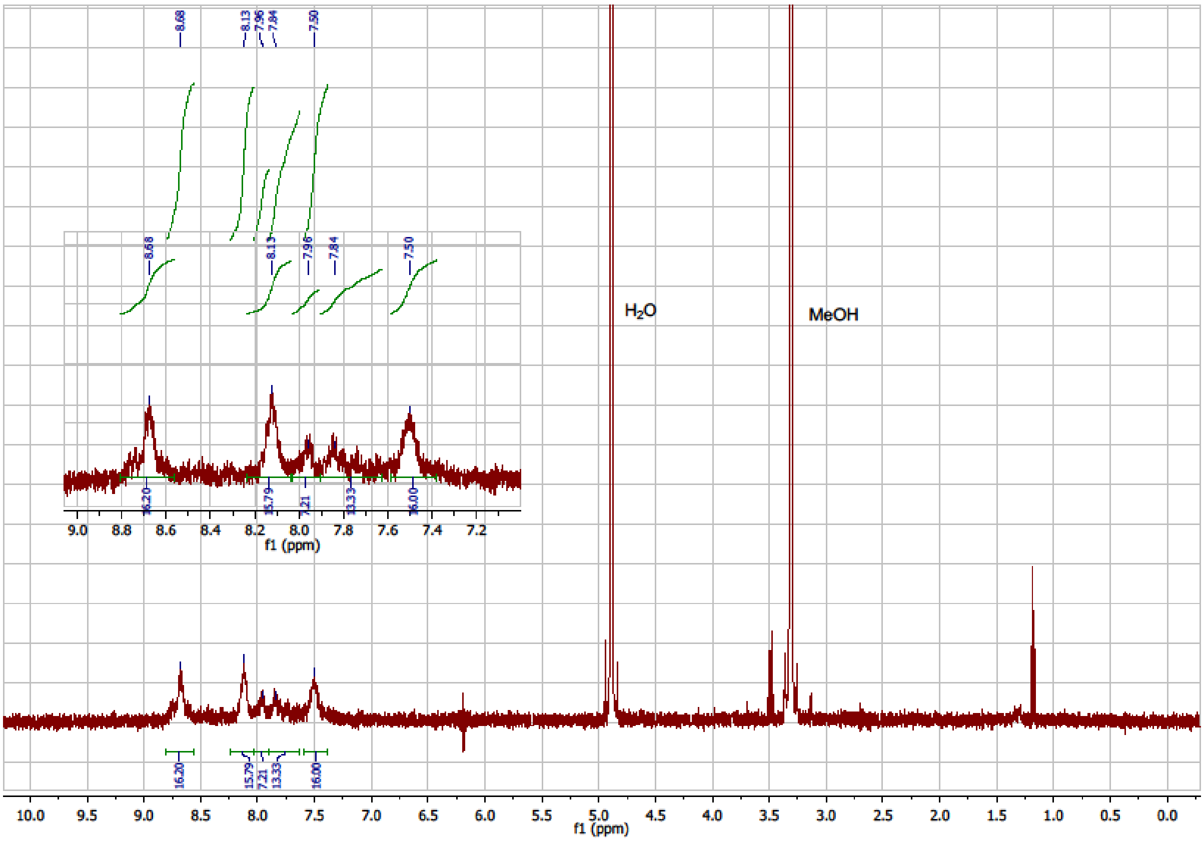

Figure S2:: ^{1}H NMR of **8**.

Mass spectrometry data

High resolution MALDI (pos) with DCTB as matrix for ligand **7**.

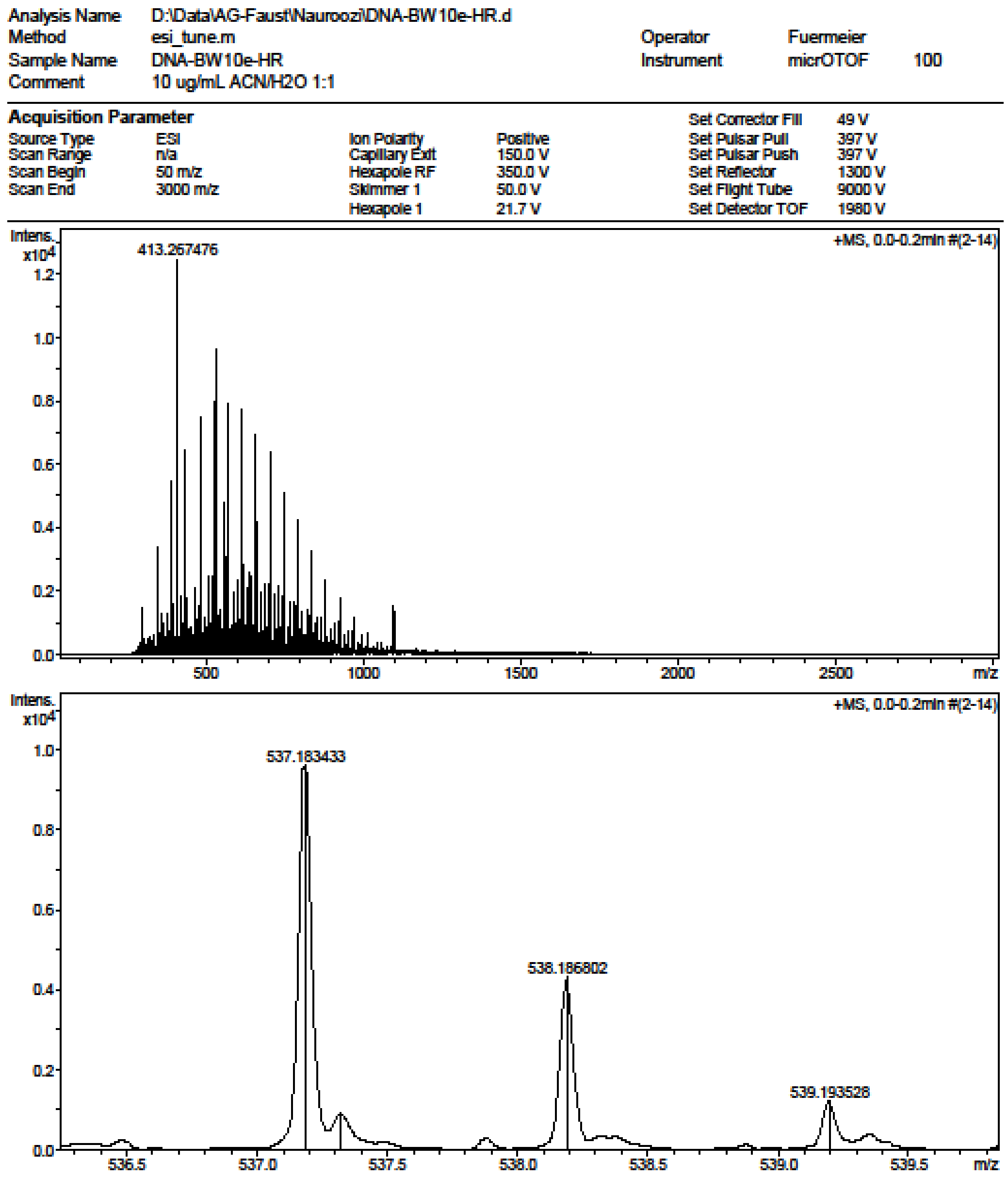

Figure S3: High resolution MALDI (pos) of **7**.

APCI $[M+H]^+$ for ligand **7**.

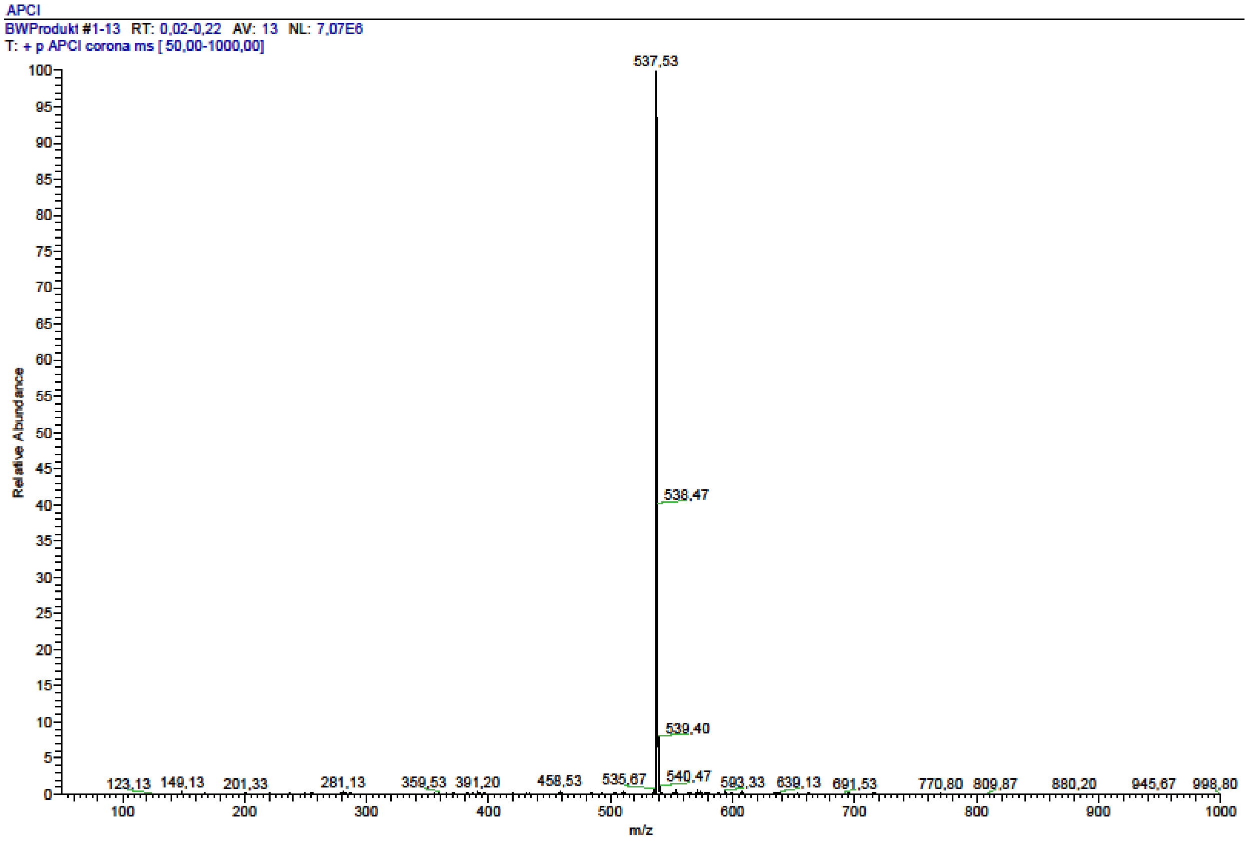

Figure S4: APCI of **7**.

High resolution MALDI (pos) with DCTB as matrix for complex **8**.

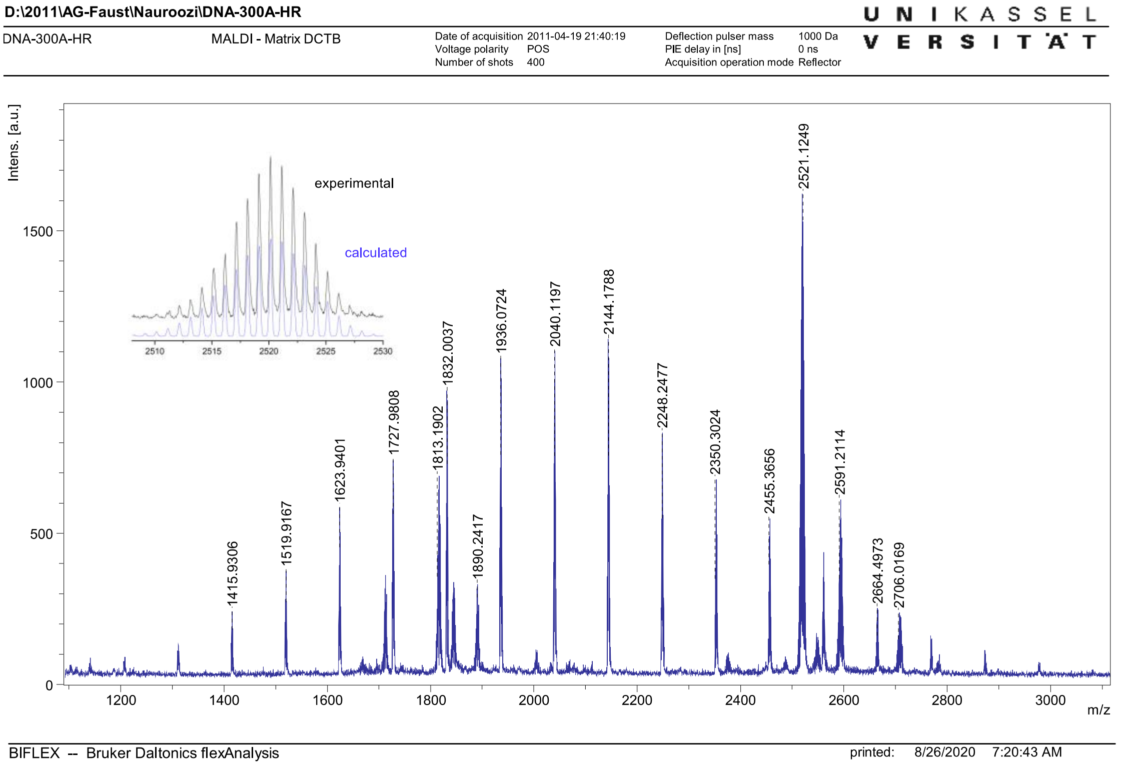

Figure S5: High resolution MALDI (pos) of **8**.

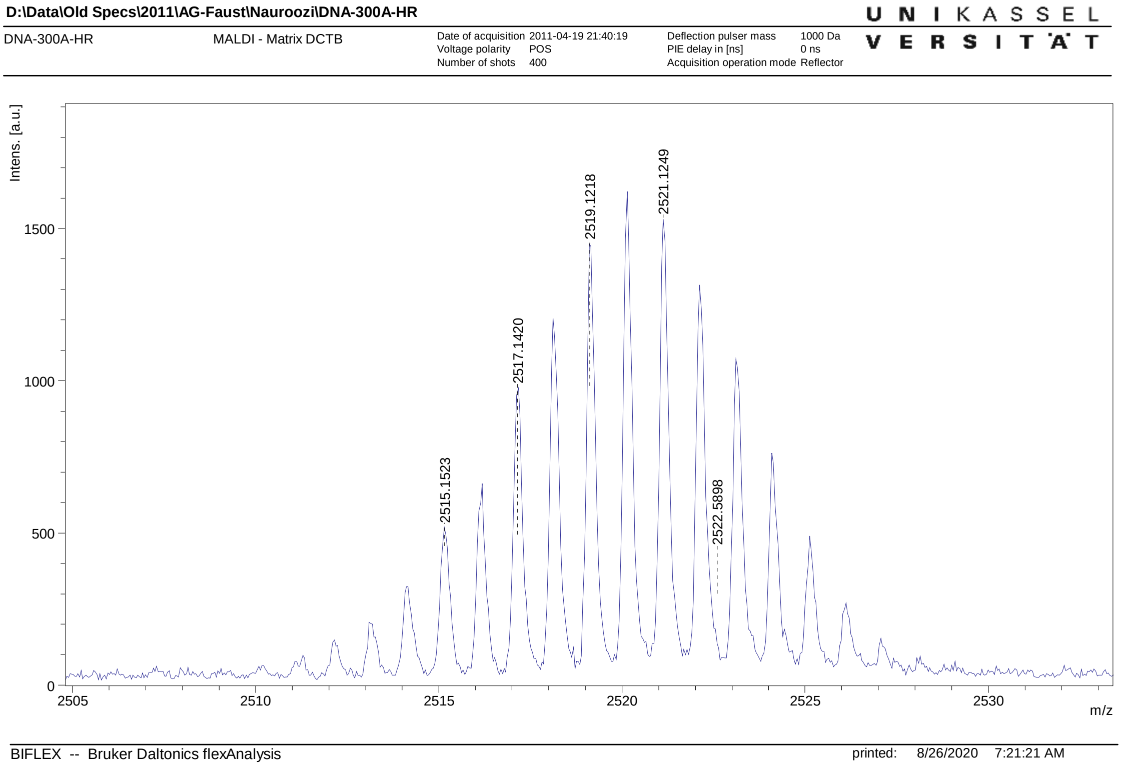

Figure S6: Isotopic pattern of complex **8**.

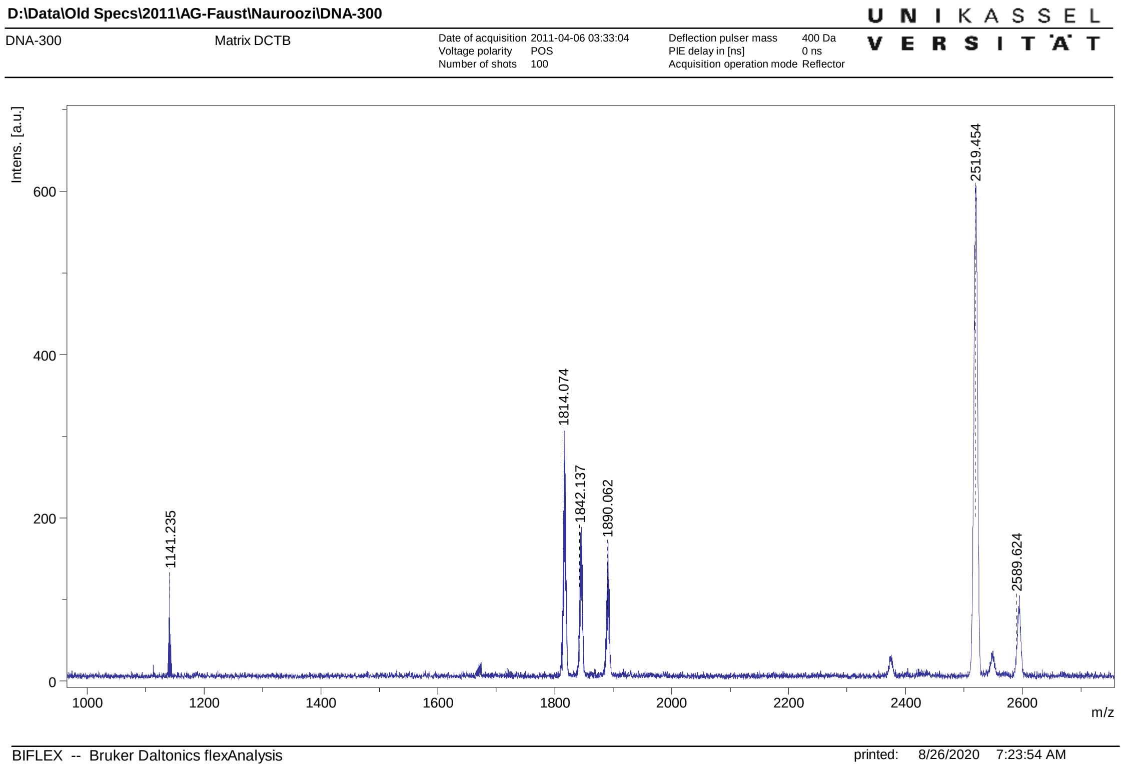

Figure S7: High resolution MALDI (pos) of **8** subtracted by signals of polymeric matrix.

UV-vis absorption spectrum of (5-ethynyl)-2,2'-bipyridine

In CH_2Cl_2 at room temperature.

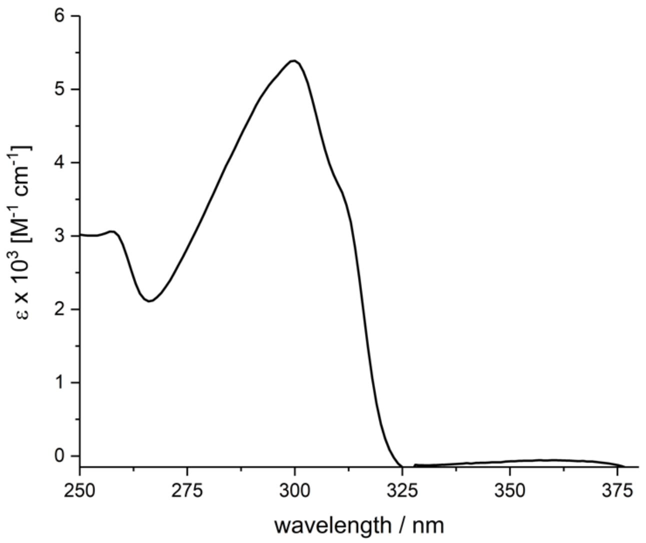

Figure S8: UV-vis spectrum of (5-ethynyl)-2,2'-bipyridine.

Cyclic voltammetry of (5-ethynyl)-2,2'-bipyridine

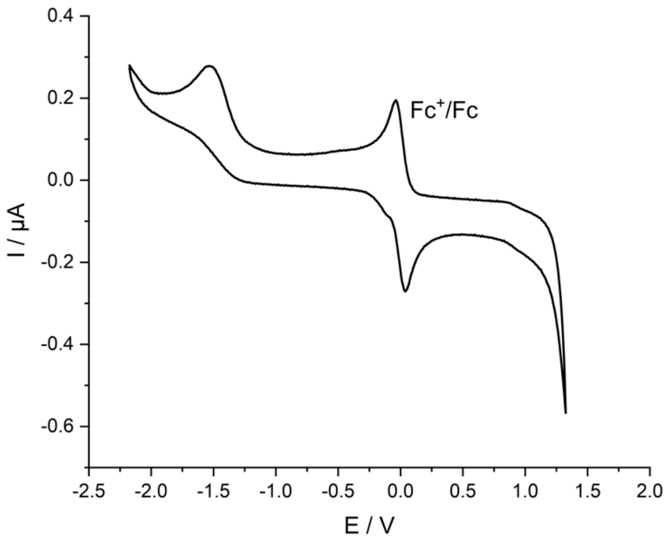

Figure S9: Cyclic voltammogram of (5-ethynyl)-2,2'-bipyridine in CH_2Cl_2 containing 0.1 M $^nBu_4NPF_6$ with Ag/AgCl as reference electrode, Pt wire as counter electrode and Pt disc as working electrode. Scan rate 150 mV s^{-1}. Data referenced against Fc^+/Fc.

Differential Pulse Voltammogram of 8

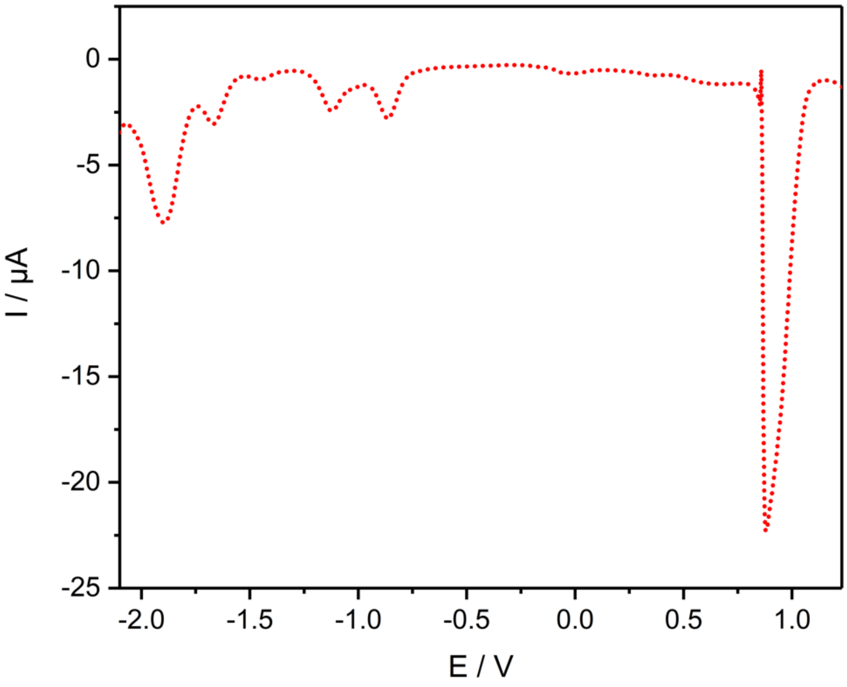

Figure S10: Differential pulse voltammogram of **8** in CH_2Cl_2 containing 0.1 M $^nBu_4NPF_6$ with Ag/AgCl as reference electrode, Pt wire as counter electrode and glassy carbon as working electrode. Scan rate 50 mV s^{-1}. Data referenced against Fc^+/Fc.

References

1 A. Boillon, J. Lancelot, V. Collot, P. Bovy, S. Rault, Tetrahedron 2002, 2885.

2 V. Grosshenny, F. Romero, R. Ziessel, J. Org. Chem. 1997, **62**, 1491.

3 A. Lützen, M. Hapke, *Eur. J. Org. Chem.* 2002, 2292-2297.
